# A Reminder for Absentees

**Published:** 1893-06-17

**Authors:** Stuart Knill

**Affiliations:** Lord Mayor. The Mansion House, London


### EDITOR'S LETTER-BOX.

[Our correspondents are reminded that prolixity is a great bar to publication, and that brevity of style and conciseness of statement greatly facilitate early insertion.]

### A REMINDER FOR ABSENTEES.

SIR,—You have in years past on the approach of Hospital Sunday, afforded the Lord Mayor the privilege of appealing on behalf of the Hospital Sunday Fund, which was established in 1872 to aid the hospitals and dispensaries of the metropolis in their beneficent work of providing medical and surgical treatment for the poor of London. It now becomes my duty on the 21st anniversary of Hospital Sunday to ask this same courtesy at your hands, and to earnestly commend the claims of the fund to the various congregations to whom appeals will be made by the clergy and ministers of religion generally next Sunday.

There are now no fewer than 181 hospitals, dispensaries, and convalescent homes claiming assistance from the Hospital Sunday Fund, and it is estimated that at least £100,000 will be required to make good the balance of deficiency which these institutions have incurred in tending upon the sick and suffering in this huge metropolis.

As the receipts on Hospital Sunday have steadily risen from £27,700 in its first year to £45,330 in 1891, I am not without hope that the collections on Sunday next will very materially recoup the large item of liability with which the medical charites have become burdened.

To aid in this great work of mercy and charity is a privilege which few would care to be deprived, and it would appear difficult to over-rate the great value and significance of this combined and simultaneous effort on the part of all creeds and faiths on this one day of the year to provide for the wants of the sick and the dying in this vast community in which we live.

To those endowed with health and strength in their families and in their homes, an appeal of this kind cannot surely be made in vain, and for very gratitude for the mercies which they have enjoyed, they will, I feel certain, respond to the call which on Sunday will be made to them.

I shall be happy to receive at the Mansion House the donations and offerings of those unable to be present at divine service in London on Hospital Sunday, but who may be willing to add their contributions to the fund.

Thanking you for your courteous assistance in publishing this appeal, I have the honour to be, Sir, your obedient servant, STUART KNILL, Lord Mayor.

The Mansion House, London, 8th June, 1893.

